# Optimization of Tensile Strength in the Paper Material Cutting Process Based on CO_2_ Laser Process Parameters

**DOI:** 10.3390/ma16072719

**Published:** 2023-03-29

**Authors:** Ivan Pincjer, Nada Miketic, Vesna Gvoic, Katarina Maricic, Djordje Vukelic, Miljana Prica

**Affiliations:** 1 Department of Graphic Engineering and Design, Faculty of Technical Sciences, University of Novi Sad, 21000 Novi Sad, Serbia; pintier@uns.ac.rs (I.P.); miketic.nada@uns.ac.rs (N.M.); kecic@uns.ac.rs (V.G.); maricic.katarina@uns.ac.rs (K.M.); 2 Department of Production Engineering, Faculty of Technical Sciences, University of Novi Sad, 21000 Novi Sad, Serbia; vukelic@uns.ac.rs

**Keywords:** laser cutting paper material, tensile strength, laser parameters

## Abstract

This paper examines the impact of the CO_2_ laser parameters on the tensile strength, which is one of the most important properties of paper packaging in the process of cutting paper material. The study was performed on a paper material sample Fbb Board/Ningbo Spark C1S Ivory Board by examination of the influence of four independent variables: paper material grammage, cutting speed, laser power, and resolution on the tensile strength by using definitive screening design. Optimum process conditions of four variables that maximize the tensile strength were predicted and validated accordingly. Results confirm that laser power, paper material grammage, and cutting speed are the main process parameters that mostly affect the tensile strength. Besides individual parameters, two statistically significant interactions were obtained: laser power and cutting speed, and cutting speed and laser resolution. Maximum tensile strength values (20.37 N/mm) were achieved using the laser power of 60.6%, cutting speed of 3.24%, resolution of 2500 Hz, and a paper material grammage of 326.85 g/m^2^. With laser power at middle values and at a lower speed, a maximum tensile strength value can be obtained. Increasing the laser power and cutting speed will produce a slight lowering of tensile strength.

## 1. Introduction

Although invented in 1960 [1], laser technology is still considered to be a new innovative tool in manufacturing industries, and the conventional manufacturing methods remain present and widely used. As a widespread technology with distinguish efficiency and accuracy, as well as a promising future, laser cutting has been extensively applied in the cutting of various materials: wood-based materials [2,3], concrete [4], cement-based materials [5,6], polymeric materials, such as polymethyl methacrylate [7,8,9], polypropylene [8], and polystyrene [10], as well as various metallic materials: stainless steel [11,12,13,14,15,16], carbon steel [17], aluminum alloy [17], and metallic plates [18]. The first use of the laser in the paper industry dates back to the 1970s, when laser was introduced for perforating cigarette paper, cutting paper, and paperboard [19]. With this development, laser technology showed considerably good results and characteristics for use in the graphic industry start to replace conventional machines over time. Additionally, laser technology enabled the development and invention of new printing techniques (laser printers) and broadened the possibilities of this industry [20]. Aside from printing, laser technology usage is increasing in postpress. Postpress (finishing) is an important and time-consuming part of the graphic industry. The use of lasers in postpress production fits perfectly into the digital print workflow. There are also material and packaging industries in which lasers completely replace mechanical blades, and lasers have become an integral part of the specific printing machines (in-line systems). The packaging industry benefited greatly regarding the quality of the laser cut during the interaction with paper material. The paper material and the number of non-metallic materials, such as glass, plastic, rubber, wood, textile, etc., are suitable for cutting with a CO_2_ laser. These materials are a big part of the packaging industry, used as the raw material or for the final product. Researchers showed several differences in comparison with conventional cutting methods and the benefits of cutting paper materials using CO_2_ laser [19,21,22]: no need to change tools if cutting different geometry and patterns, contact-free method, higher cutting speed, smaller amounts of dust, and reduced material lost.

The conventional paper material cutting process is done by application of shear force to achieve separation of the fibers. Cutting occurs when ultimate shearing strength of the paper material is exceeded [23]. Cutting is an important component of the printing process, as the cutting precision directly affects the precision of print and the quality of the finished product in the packing industry [24].

The basic principle of laser cutting of paper materials is responsible for the number of advantages during the cutting process. Focusing laser rays from the resonator on the paper material surface causes the heating of the surface and chemical decomposition to occur [25]. The chemical degradation products of the material evaporate or are removed from the cut kerf with laser gas assistant [25].

When it comes to manufacturing the packaging for products, there are several crucial requirements. Factors that stand out are the tensile strength of the paper material and the quality of the cut edge [26]. Those factors are responsible for the mechanical quality of the packaging, and if these factors are loose, the packaging production and the final product can be threatening and even impossible to produce [27]. Paper dust and fibers sticking out of the material, produced by mechanical blades, can be the cause of severe problems in the packaging and papermaking industry [28]. Water can also enter through the exposed cut edges of packaging [27]. For example, in the liquid packaging industry, this problem should be avoided because liquid absorption properties of the cut edge are very important for minimal edge-wicking of the product [28]. Many studies confirm the advantages brought by laser technology in the papermaking and packaging industry. CO_2_ laser technology ensures a sealed and sleek cut edge without sticking out fibers and minimizing dust occurring as a by-product [28]. Regarding this, researchers [28] made scanning electron microscopy (SEM) images that clearly show the difference in structure between mechanical and laser-cut edges. The development and creation of innovative and complex packaging designs became possible with the implementation of laser [19]. For example, laser-cut openings are very easy to tear, and they fulfill the aseptic demands of packages [19]. Additionally, laser-cut openings can be done in places where it is impossible to use traditional techniques [19]. The perforation of paper materials using conventional methods leads to loss of the material strength caused by ripped fibers and uneven size of the holes, and some holes can be closed or not fully perforated [29]. In contrast, when laser technology is used to create perforation, the holes created are all even and open [29].

Paper material is proven to provide the best cutting results during the interaction with the CO_2_ laser owing to the highest absorbance of the cellulose molecules on the wavelength of 10.6 µm (10,604 nm), which is the wavelength of the CO_2_ laser [29]. However, there are many influential factors affecting the cutting process. Previous studies analyzed these influential factors such as moisture content in the paper material, coating materials, fillers, printing ink, grammage, thickness, volume, etc. In the previous period, paper material cutting was investigated in different ways. Hovikorpi et al. [30] tested the impact of different laser parameters (power, focal point position, and focal length) on the cut edge quality. The cut edge quality and kerf width were stable for each grammage and paper material type produced with parameter combinations on cutting levels. Federle and Keller [31] determined that when the power of the laser increases, the increase in the quality of the cut occurs. However, if the cutting is performed with a higher amount of strength than is required, it can result in a wider kerf and increased carbonization of the cut edge with a higher fume emission. Malmberg et al. [32] concluded that the volume of the paper material affects the speed of laser cutting. With the increase of volume, paper materials have a smaller density, and therefore a small amount of material evaporates. Consequently, the speed of cutting is increasing. Stepanov et al. [22] investigated the influence of material moisture content. The results confirmed that more laser power is required for with higher moisture content of the material, and the opposite. Malmberg et al. [33] confirmed that cutting materials with coating could result in colored edges and the production of smoke, which depends on the quality of the coating. Furthermore, the power required for cutting increases with an increasing amount of coating. Additionally, it was noticed that coating causes widening of the kerf.

Stepanov, Piili, and Salminen [34] investigated the color change of the paper material kerf after interactions with the CO_2_ laser beam. The results were inconclusive because significant color change was observed with both high and low laser power [34]. Stepanov et al. [35] used pre-calculated laser power to eliminate energy oversupply. The calculation took into account paper material grade, sample thickness, and specific heat of the paper material. In conclusion, they suggested that more accurate calculation of cutting energy is necessary, as well as the determination of the range of cutting energy needed for higher quality cuts [35]. Spicar-Mihalic et al. [36] used the pulsed CO_2_ laser system for the fabrication of paper-based microfluidic devices. Cutting through full depth of the paper material was accomplished by increasing the power or decreasing the speed of the laser head. The smallest standard deviation of the cutting was accomplished with the smallest power used for a given speed settings [36]. Mahmud et al. [37,38] created compact paper-based devices using CO_2_ laser. By adjusting the power and speed of the laser head, they fabricated low-cost microfluidic glucose test devices [37,38]. Microfuidic paper-based analytical devices were successfully produced by laser cutting microstructures with the smallest hydrophilic strip of 0.5 mm, which indicates the high precision of the laser-cutting technique [39].

Pillai et al. [40] demonstrated the simplicity of laser-cutting technology by constructing a reasonable cost mobile solar powered desktop laser-cutting machine for fabric and paper material. Pages et al. [41] investigated paper material laser cutting by using a single emitter laser diode, within a desktop printer. Results demonstrated that paper material with a suitable sorbing ink can be cut with a laser diode. Easy tear-able and folding lines can be made by increasing the speed. Huppert et al. [42] presented a system with a laser cutter which allows for hand-sketched objects on a paper material notebook and directly sends the cutting data to a laser cutter for manufacturing tangram puzzles. Happonen et al. [43] stressed the shortage of well-known and widely available published research articles and measurement data with actual cut speeds with high quality cut edges. The laser cutting of paper material has proven to have a strong potential for industrial application, and if there were more data, laser cutting could potentially substitute for conventional paper material cutting methods in various application in related industries [43].

Previous studies have not explored the optimization and impact of laser process parameters (power, speed, resolution) and paper material grammage on the tensile strength during the paper-cutting process. By innovating the optimization of the paper material cutting process, the optimal settings for manufacturing paper-based products with higher mechanical strength, durability, and overall quality can be ensured. This, in turn, could lead to more efficient and cost-effective manufacturing processes for these products, benefiting a wide range of industries that rely on paper-based packaging materials.

To understand the importance of tensile strength for paper and paperboard, it is necessary to become familiar with the various printing techniques to which paper and paperboard are subjected, as well as the indispensable use of paperboard in the packaging industry. For printing paper, high tensile strength is required to withstand the forces that are applied to the paper as it is fed through the press. In offset printing, for one of the most commonly used printing processes, whether sheet-fed or web-fed (newspaper printing), the paper material is subjected to various forces for which high tensile strength is required. In addition, paperboard is used for packaging, which is extremely important for products from various industries. Here, the paperboard is subjected to even more stresses. A product is packed in a package to protect it and transport it safely, and to facilitate handling during transportation and use.

In contrast to previous studies, and due to the lack of information on laser cutting of packaging paper material, this study aims to investigate the optimization of CO_2_ laser process parameters (power, speed, and resolution) on different paper material grammages that optimize tensile strength during the cutting process of paper material. The experimental process conditions were optimized and verified with a new generation of design of experiments, and a definitive screening design (DSD).

## 2. Materials and Methods

**Figure 1:**
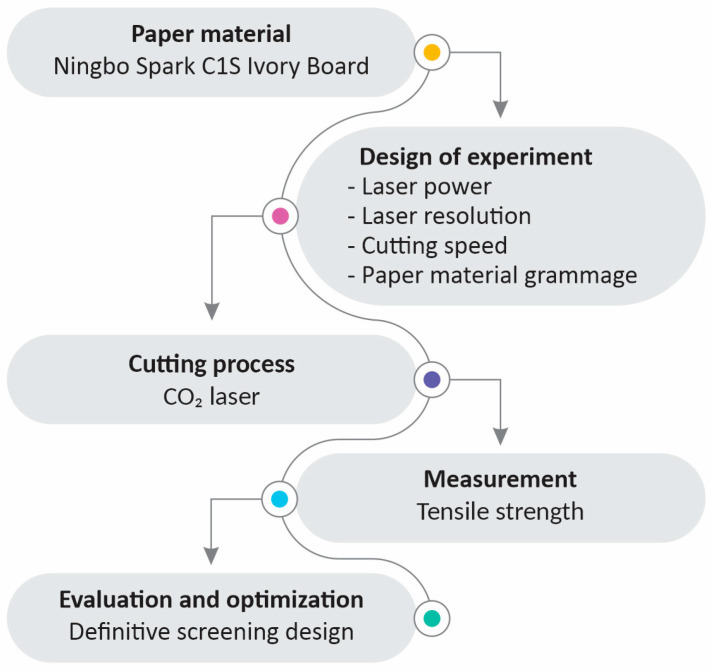
Research methodology.

The research methodology is shown in Figure 1.

Different process parameters were analyzed to find the optimal adjustment of laser parameters for cutting paper material—paperboard (Ningbo Spark C1S Ivory Board, Zhonghua Paper Ltd., Ningbo, China). This material is usually used for pharmaceuticals packaging, chocolate, and confectionery packaging, and the base board for polyethylene coating is used for frozen food packaging, book covers, shopping bags, media packaging, and tea bags. The bulk and stiffness of this paper material are equivalent to best GC boards, they are aesthetically pleasing, and the pigment coating on the backside improves printing potential compared to manila back. In addition, this paper material provides optimal performance in the high-speed conversion process and excellent performance on folding, die-cutting, and scoring. The technical information for the investigated paperboard samples is presented in Table 1.

The research examined the influence of four independent variables, namely paper material grammage, cutting speed, laser power, and resolution on tensile strength. The selected levels of independent variables are shown in Table 2. The levels of laser process parameters are selected according to the laser manufacturer’s recommendations depending on the type of material to be cut. In Table 2, the units for laser power and cutting speed are given in %, as they are set accordingly in the laser.

**Figure 2:**
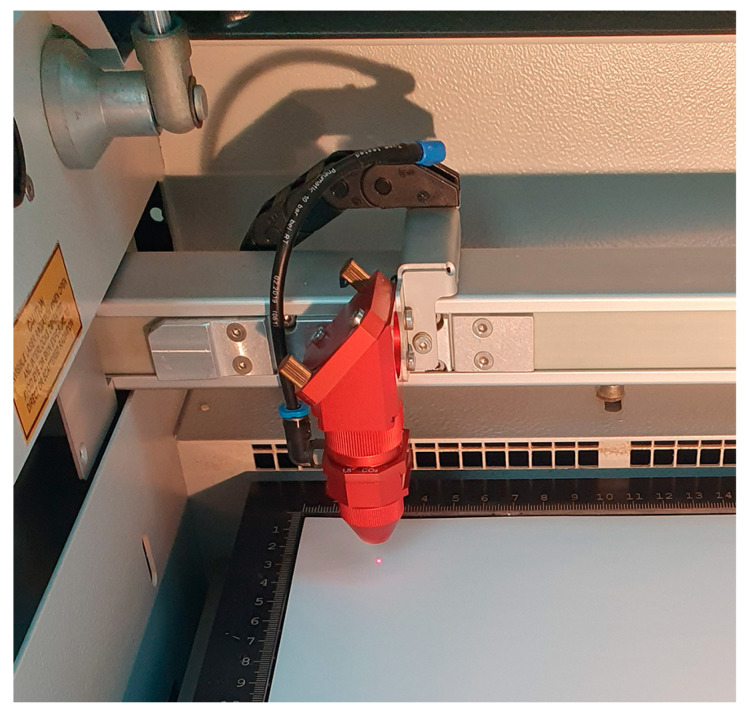
Laser cutting process.

Samples were cut using Trotec Speedy 300 (Trotec, Marchtrenk, Austria), CO_2_ laser (Figure 2), with power 80 W and maximum cutting speed 3.55 m/s, which produces a laser beam with a wavelength of 10.6 µm. The laser is equipped with a 1.5-inch lens, and it can be used for cutting, engraving, and marking. Movement on the laser head is possible in XY direction controlled by software JobControl Laser Software (v 11.1.1). The software enables total control over the laser device, including head position, the focus of the beam, and all other parameters. The Trotec Speedy 300 device was equipped with suction, which removes fumes produced during laser beam and material interaction. The cutting operation can be done with paper materials, acrylic < 6 mm thick, and wood < 8 mm thick. Utilizing the managing software JobControl^®^, it is possible to program the parameters for the job and choose adequate material.

**Figure 3:**
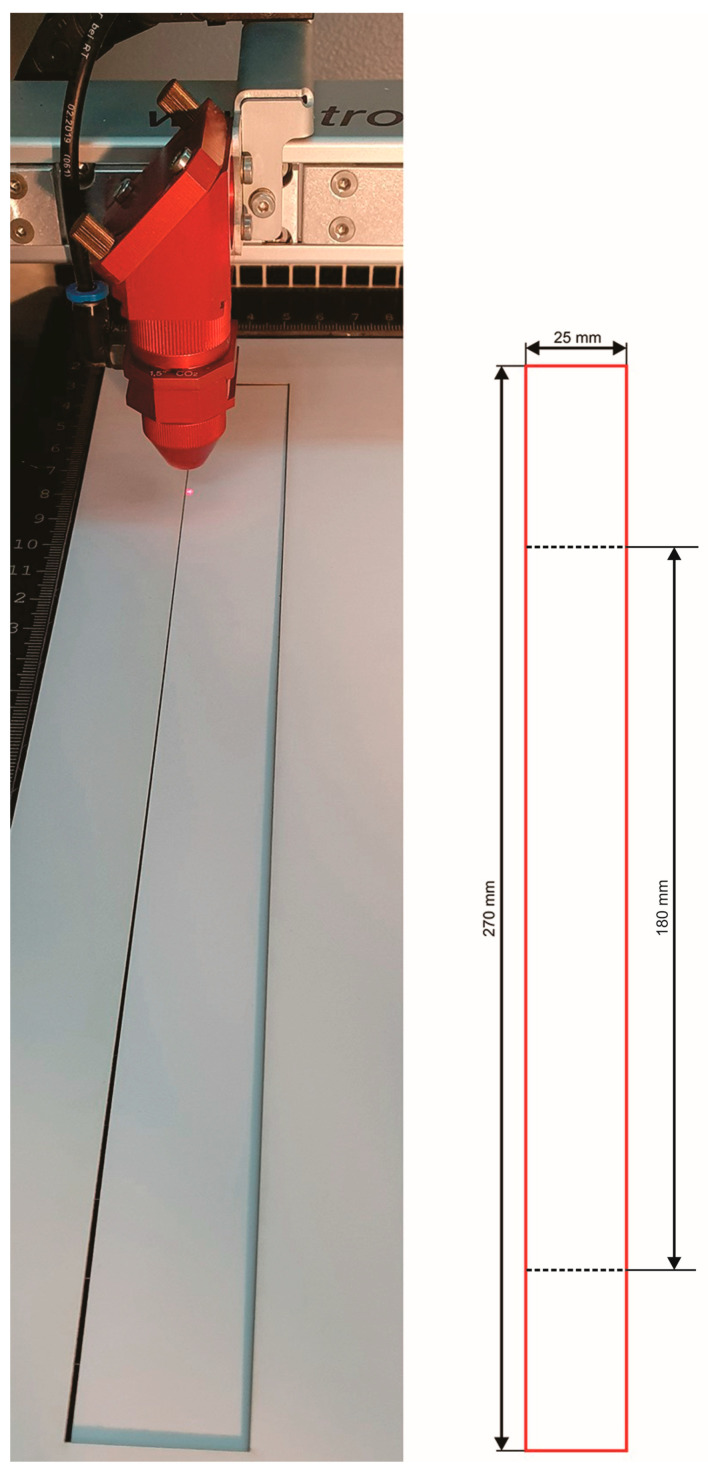
Experimental specimen.

Experimental specimens (Figure 3) were cut using a laser with dimensions according to the standard for the tensile strength testing recommended by TAPPI T 494-om-1. Specimen dimensions have a width of 25 mm and have a crosshead height of 180 mm. The thickness of the paper material with the following grammage of 295 g/m^2^, 325 g/m^2^, and 360 g/m^2^ are 0.56 mm, 0.58 mm, and 0.60 mm, respectively.

**Figure 4:**
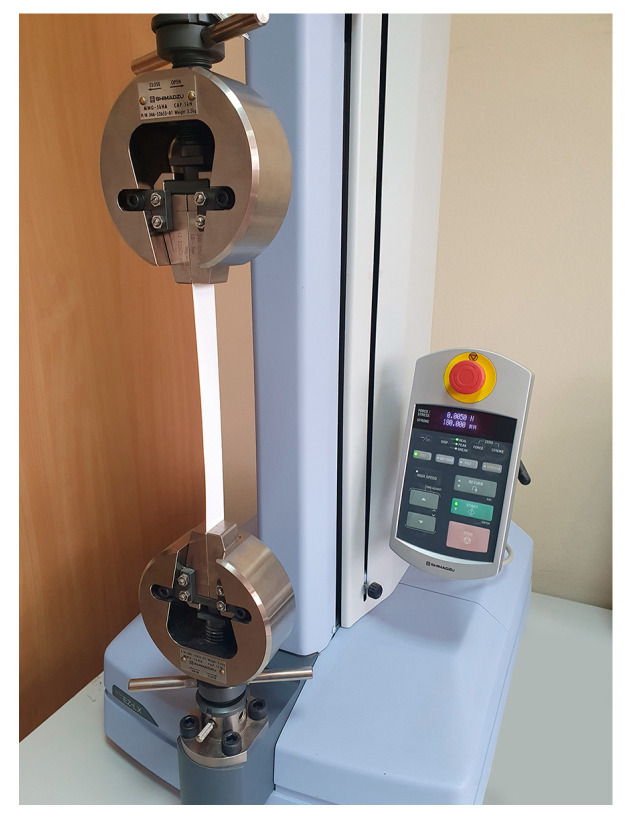
Tensile strength testing.

Tensile strength testing was conducted using Shimadzu Compact Tabletop Testing EZ-LX (Shimadzu, Kyoto, Japan), (Figure 4). Specimens were tested utilizing the load cell type of 2.5 kN and a crosshead speed of 25 ± 5 mm/min.

The investigation and optimization of the process, specifically paper material cutting using laser technology, requires a large number of experiments consisting of several process parameters that impact the material. Therefore, a well-considered statistical approach was necessary to apply in order to select the most important operating parameters for the screening design. The design of the experiment in this study is based on the DSD [44,45,46]. Due to a number of advantages over the traditional separation and screening experiments, the DSD method became widely used. The main importance of DSD is the possibility of decreasing the number of the required experiments to assess the main effects, quadric effects, and two-factorial interactions. Furthermore, the DSD method significantly decreases the number of experiments while maintaining maximal precision, which lowers the costs and materials during the experiment execution. This is possible due to introducing the fake factors, which, as a result, provide more factors than required in order to gain higher statistical power of the experiment, while these fake factors are not included in the analysis. DSD method requires a minimum of 2k + 1 run (k represents the total number of process parameters examined). As shown in the Jones and Nachtsheim study [47], when the number of factors is even, as is the case with this study, adding two fake factors, at a cost of four additional runs, increases the efficiency of the design. In addition, two extra center runs were added to get a better estimation of pure error (experimental error) as part of the estimate of the error variance. Thus, in this case, the basic design used 6 × 2 + 1 = 13 runs (with the two extra columns dropped), which, with an added replication and two extra center runs, made for a total of 28 runs.

## 3. Results and Discussion

### 3.1. Evaluation of the DSD Model

In order to characterize the system influenced by a variety of process conditions on the tensile strength of tested paper material, DSD statistical analysis is used. According to the basic DSD schema of the experiment (Table 3), 13 experiments conducted in duplicate with the addition of two central points provided 28 experiments. The results obtained by tensile strength measurement are presented in Table 3. The tensile strength is defined in N/mm according to standard ISO 1924-3:2005.

It is important for the material to maintain a tensile strength that is as high as possible during the phase of cutting process. Based on the obtained results, different values of tensile strength were measured under different combinations of process conditions, which confirms the hypothesis that the tensile strength of the paper is dependent on different process conditions. In the case of several experiments (4, 6, 12, 17, 19, and 25), cutting did not occur due to the minimum laser power and high cutting speed. When the laser hits the paper at the lowest power, it must stay in the same place longer to avoid damaging the paper structure. If the speed of the laser is high at minimum power, the laser beam will not penetrate deep enough into the structure of the paper and cutting will not occur. To cut the paper with the minimum power of the laser, the speed must be slowed down, as the results show.

Table 4 shows the values of initial tensile strength and descriptive statistical parameters for different paper material grammages and for all experiments. The minimum value of tensile strength 12.106 N/mm was obtained for the maximum value of laser power 90%, minimum cutting speed 1%, mean value of laser resolution 2500 Hz, and minimum value of paper material grammage 250 g/m^2^. The maximum value of tensile strength 20.467 N/mm was obtained for the mean value of laser power 55%, the maximum value of cutting speed 8%, the maximum value of laser resolution 3000 Hz, and the maximum value of paper material grammage 360 g/m^2^. The lowest dispersion of results (minimum standard deviation) was obtained for the mean value of paper material grammage 325 g/m^2^. The high value of the ratio (between the maximum value and the minimum value of the tensile strength) of 1.691 for all experiments shows that the tensile strength can be controlled in a wide range. With a different combination of input parameters, the tensile strength can be significantly influenced depending on the needs of the specific operating conditions of the paper material. In addition, the obtained experimental results indicate that different tensile strengths are obtained with the same paper material grammage and different laser parameters (power, cutting speed, and resolution). This indicates that microstructural changes occur at the cut edge and that the mechanical properties of the material change.

Despite the software proposition of several statistical models which provides a good approximation of the obtained results, the final model selection is based on standard selection criteria: coefficient of determination (R^2^), adjusted coefficient of determination (R^2^adj), Bayesian Information Criterion (BIC), Akaike Information Criterion (AIC), Root Mean Square Error (RMSE), analysis of variance (ANOVA), and diagnostic diagrams. Table 5 presents a summary of fit, while the results of the ANOVA test and “lack of fit” test are given in Table 6. The accuracy of the model selection is confirmed by the coefficient of determination (R^2^ = 0.937), which implies that 94% of the variance is explained with an independent variable, whereas only 6% of the total variance did not cover using this model. The simplicity of the model, good approximation, and elimination of inappropriate data filtering is demonstrated by the lowest AIC and BIC parameters. The standard deviation of the residuals, which provides information about the concentration of data around the best fit line, is represented by RMSE, where a small value indicates a good fit and accuracy of the model prediction. ANOVA was conducted in order to investigate statistical significance between input variables. Based on the obtained value of the parameter F < 0.0001 (Table 6), a statistical significance of the regression model was established. Furthermore, the validity of the chosen model was additionally proven with the insignificance of the “lack of fit” test (F > 0.05).

**Figure 5:**
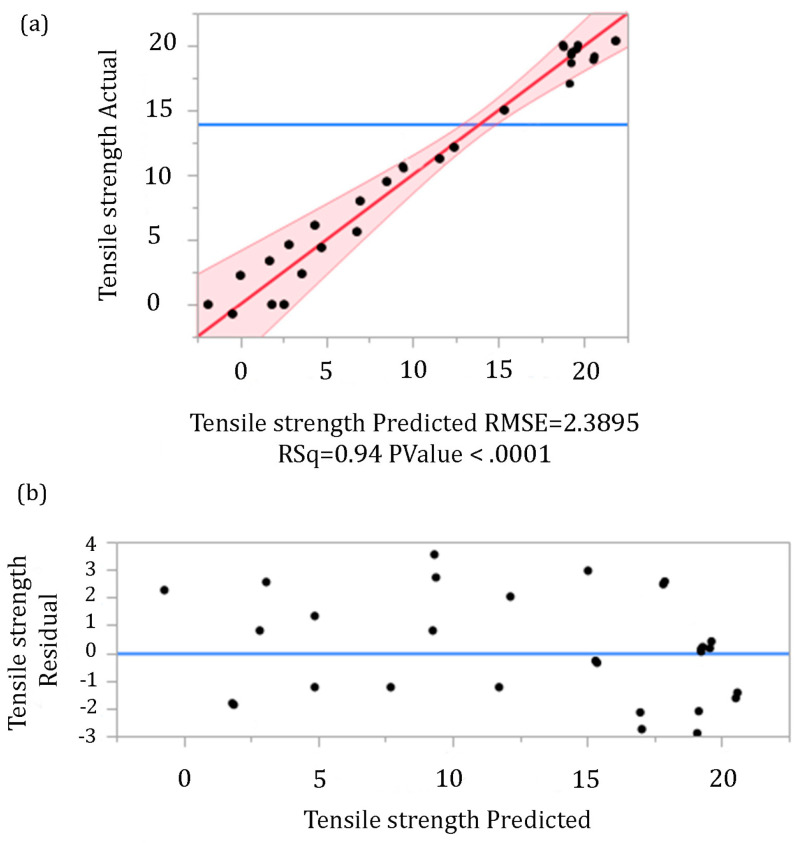
Diagnostic plots, (**a**) actual vs. predicted values of tensile strength; (**b**) deviation diagram of standardized residuals in relation to the zero line.

Additional testing of the adopted model adequacy was conducted using diagnostic plots (Figure 5). A scatter plot of actual vs. predicted tensile strength values (Figure 5a) shows that the points closely follow the regressed diagonal line without outliers. On a deviation diagram of standardized residuals, there is no tendency for value scattering, and dots are randomized in space, implying that the regression model has a good representation of the investigated model (Figure 5b).

### 3.2. Process Optimization

Based on the estimated parameter values and their probability level (Table 7), it was concluded that laser power, paper material grammage, and cutting speed are the main process parameters that mostly affect the tensile strength. Besides individual parameters, two statistically significant interactions were obtained: laser power and cutting speed and cutting speed and resolution.

**Figure 6:**
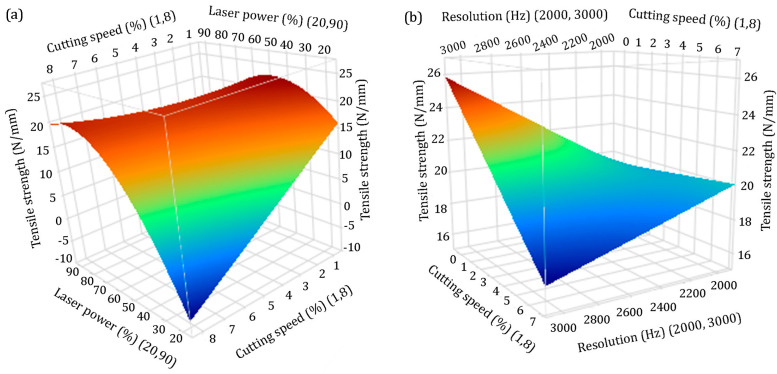
3D response surface plots of statistically significant interactions, (**a**) laser power and cutting speed, (**b**) cutting speed and resolution.

Laser power has an important role in ability of the regression model which explains the behavior of the observed process. The positive sign in front of the linear member suggests that increasing the laser power will contribute to the increase of tensile strength only to one point, after which further increases have an adverse effect on the final specimen strength. This influence can also be noticed in the optimization diagram (Figure 6), where the optimum laser power of 60.6% is in the middle of the central and top borders.

Although not statistically significant, laser resolution is a statistically significant interaction with cutting speed. The second statistically significant interaction exists between laser power and cutting speed. The statistically significant interactions are illustrated in 3D response surface plots shown in Figure 6.

In Figure 6a, it is shown that at a constantly high level of cutting speed (8%), laser power has a pronounced influence on the tensile strength increment, while the greatest influence is noticeable at the maximum level of laser power (90%). On the other hand, when the laser power is at a constantly low level (20%), cutting speed has the greatest influence on the tensile strength at its lowest values (1%).

Figure 6b illustrates significant two-way interaction between cutting speed and laser resolution. At the constant high laser resolution values, tensile strength increases with a decrease in cutting speed. It can be concluded that the influence of resolution on the increasing tensile strength is greater when the resolution value is set and held constant at 3000 Hz.

**Figure 7:**
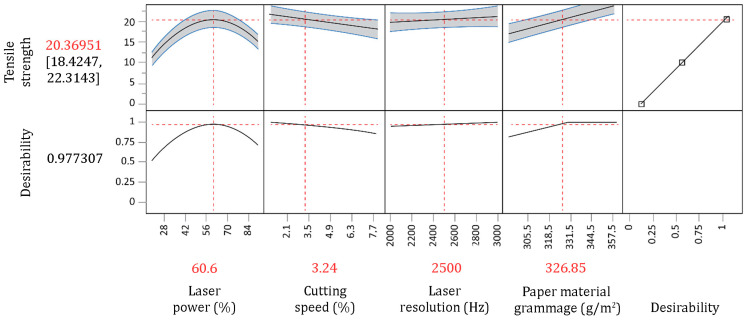
Optimization diagram.

The optimized process parameters, which provide maximum tensile strength, are presented in the optimization diagram in Figure 7. The statistical model proposes that maximum tensile strength values (20.37 N/mm) are achieved using the laser power of 60.6%, cutting speed of 3.24%, resolution of 2500 Hz, and a paper material grammage of 326.85 g/m^2^.

Furthermore, the influence of individual process parameters on the tensile strength can be explained by observing four cells independently, which illustrates how tensile strength changes as a function of one variable while all other variables remain constant. According to Table 7 and Figure 7, increasing the laser power up to a certain point increases the tensile strength, while further laser power increase contributes to the adverse effect on the observed variable. The explanation of this phenomenon can be related with dual-phase mechanism which occurs with the increase of laser power, where a plasma plume explosion contributes to the scatter of laser beam, which leads to the enlargement and damage of the cut kerf [25]. In addition, it was established that tensile strength decreases with the increase of cutting speed. By increasing the cutting speed, the laser beam affects the paper material for a shorter time and passes over it faster with a constant power, so its impact on one point is shortened. In order to obtain efficient cutting, the laser power must be increased. However, increasing the leaser power leads to more significant damage of the paper material and the tensile strength decreases. Otherwise, if the laser power is not increased, the cutting will be incomplete, which results in the tensile strength decreasing [43]. In order to explain this phenomenon, a detailed investigation must be conducted in the future, following the assumption that the fibers on the edge of the paper material are “welded” under the influence of heat and thus create a sealed edge around the entire paper material sample that increases its tensile strength. Moreover, results pointed out that an increase of laser resolution and paper material grammage, separately, lead to the increase of tensile strength. Laser resolution increase causes the increase of laser pulse density that affects paper material. Due to the higher pulse density, the laser power does not have to be increased, and therefore the damage of the paper material is less. If the resolution decreases, in order to obtain efficient cutting, the laser power must increase, which leads to damaging of paper material. The obtained result corresponds to the hypothesis that the appropriate pulse density completely welds the edges and thus increases the tensile strength. Ultimately, the increase of paper material grammage leads to the increase of tensile strength as a result of greater thickness of the paper material, which is stiffer, stronger, and more difficult to bend.

**Figure 8:**
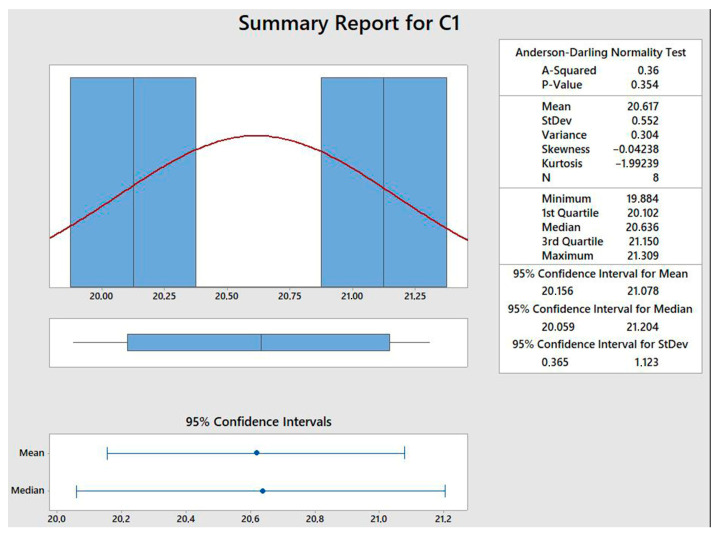
A 95% confidence interval.

In order to verify the validity of the adopted regression model in terms of process optimization, a verification of proposed optimal process parameters was conducted with eight additional runs (Table 8). Based on the 95% confidence interval (20.16–21.08 N/mm), which is presented in Figure 8, it can be concluded that the proposed optimized tensile strength of 20.37 N/mm fits in the confirmatory run confidence interval, confirming the adequate selection of the regression model.

## 4. Conclusions

The objective of this study was to investigate the influence of CO_2_ laser process parameters (power, speed, and resolution) and paper material grammage on the tensile strength during the paper-cutting process. The experimental process conditions were optimized and verified with a new generation of experimental design, a definitive screening design (DSD) which provided insight into the significant main process parameters and two-way interactions:

- Laser power, paper material grammage, and cutting speed are the main process parameters that affect the tensile strength of the paper material. Increasing the laser power increases the tensile strength up to a certain level, while after that it has an inverse effect on the final specimen strength.
- The interaction between laser power, laser resolution, and cutting speed was found to be statistically significant. At a constant high cutting speed (8%), the laser power has a pronounced influence on the increase of tensile strength, while the greatest influence was observed at the maximum laser power (90%). At constant high values of laser resolution, the tensile strength increases with decreasing cutting speed. The tensile strength is greater when the resolution value is set to 3000 Hz and kept constant.
- The statistical model states that the maximum tensile strength values (20.37 N/mm) are obtained with a laser power of 60.6%, a cutting speed of 3.24%, a resolution of 2500 Hz, and a paper material grammage of 326.85 g/m^2^. With medium laser power and a lower speed, a maximum tensile strength value can be obtained. Increasing the laser power and cutting speed results in a slight decrease in tensile strength.

This paper provides a successful insight into the methodology for determining the optimal parameters of CO_2_ laser cutting, regardless of type of paper, grammage, and laser manufacturer. The developed methodology allows for the prediction of optimal cutting parameters without large expenses.

In this paper, the effect of CO_2_ laser parameters on the tensile strength of paper material is limited to four independent influencing variables: paper material grammage, cutting speed, laser power, and laser resolution on the tensile strength using definitive screening design. In addition, the results are related to the investigated experimental conditions and one output parameter.

To overcome the aforementioned limitations, future research in the field of CO_2_ laser cutting of paper materials will focus on including more variables and materials into experiments to achieve deeper and more nuanced optimization. This could include studying the effects of different laser parameters on a broader range of materials, as well as the effects of factors such as material thickness, density, composition, etc. With a deeper understanding of the factors that affect the CO_2_ laser cutting process of paper materials, it may be possible to achieve even greater improvements in production efficiency and product quality while reducing costs. Additionally, SEM analysis and microstructure examination of cutting edge will be one of the directions of future research.

## Figures and Tables

**Table 1:** Paperboard technical information.

Property	Unit	Value
Moisture	%	6.5 ± 1.0
Smoothness PPS (Top)	μm	1.4
Brightness ISO (TOP)	%	93.0 ± 2.0
75°Gloss (Top)	%	50 ± 10
IGT Blister (Top)	m/s	≥1.5
COBB Test (60 s) (Top/Back)	g/m^2^	40 ± 15
PLY bonding	J/m^2^	≥130
Squareness	mm	≤2.0
Size Deviation	mm	0–2

**Table 2:** Independent process variables with experimental levels.

Variables	Minimum Level	Middle Level	Maximum Level
Laser power (%)	20	55	90
Cutting speed (%)	1	4	8
Laser resolution (Hz)	2000	2500	3000
Paper material grammage (g/m^2^)	295	325	360

**Table 3:** DSD experimental design and responses.

Exp. No.	Laser Power (%)	Cutting Speed (%)	Laser Resolution (Hz)	Paper Material Grammage (g/m^2^)	Tensile Strength (N/mm)
1	55	8	3000	360	20.467
2	55	1	2000	295	14.282
3	90	4	2000	360	19.898
4	20	4	3000	295	–
5	90	1	2500	295	12.106
6	20	8	2500	360	–
7	90	8	2000	325	19.155
8	20	1	3000	325	19.173
9	90	8	3000	295	15.016
10	20	1	2000	360	20.037
11	90	1	3000	360	17.050
12	20	8	2000	295	–
13	55	4	2500	325	19.510
14	55	8	3000	360	20.311
15	55	1	2000	295	14.830
16	90	4	2000	360	20.050
17	20	4	3000	295	–
18	90	1	2500	295	12.873
19	20	8	2500	360	–
20	90	8	2000	325	18.898
21	20	1	3000	325	19.164
22	90	8	3000	295	15.028
23	20	1	2000	360	19.736
24	90	1	3000	360	16.196
25	20	8	2000	295	–
26	55	4	2500	325	19.38
27	55	4	2500	325	19.290
28	55	4	2500	325	18.651

**Table 4:** Descriptive statistical parameters of experimental tensile strength results.

Parameter	Paper Material Grammage (g/m^2^)			All Experiments
	295	325	360	
Minimum	12.106	18.651	16.196	12.106
Maximum	15.028	19.510	20.467	20.467
Mean	14.023	19.152	19.218	17.777
Standard Deviation	1.242	0.271	1.634	2.612
Ratio	1.241	1.046	1.264	1.691

**Table 5:** Summary of fit.

Descriptive Factor	Value
R^2^	0.937
R^2^adj	0.906
AIC	154.371
BIC	152.525
RMSE	2.390

**Table 6:** ANOVA and lack of fit test.

Source	Degrees of Freedom	Sum of Square	Variance	F Parameter
Model	9	1530.331	170.037	29.779
Error	18	102.779	5.710	Prob > F
C. Total	27	1633.109	–	<0.0001
Lack of Fit	16	102.46285	6.40393	40.537
Pure Error	2	0.31595	0.15798	Prob > F
Total Error	18	102.77880	–	0.063

**Table 7:** Estimated coefficients of the significant main parameters and two-way interactions.

Term	Estimated Value	Standard Error	*t* Value	Probability > |t|
Laser power (%) * Cutting speed (%)	6.256	0.664	9.420	<0.0001 *
Laser power (%)	4.408	0.534	8.250	<0.0001 *
Paper material grammage (g/m^2^)	3.481	0.534	6.510	<0.0001 *
Cutting speed (%)	–2.829	0.534	–5.290	<0.0001 *
Cutting speed (%) * Resolution (Hz)	–1.837	0.738	–2.490	0.0228
Laser power (%) * Resolution (Hz)	1.194	0.664	1.800	0.0891
Resolution (Hz)	–0.224	0.534	–0.420	0.6799

**Table 8:** Experimental verification of the optimized process.

Run	Laser Power (%)	Cutting Speed (%)	Laser Resolution (Hz)	Paper Material Grammage (g/m^2^)	Tensile Strength (N/mm)
1	60.6	3.24	2500	325	20.884
2	60.6	3.24	2500	325	21.197
3	60.6	3.24	2500	325	20.071
4	60.6	3.24	2500	325	21.007
5	60.6	3.24	2500	325	20.196
6	60.6	3.24	2500	325	20.362
7	60.6	3.24	2500	325	20.911
8	60.6	3.24	2500	325	21.309

## Data Availability

Not applicable.

## References

[1] Powell J. (1988). CO_2_ Laser Cutting.

[2] Ružiak I., Igaz R., Kubovský I., Gajtanska M., Jankech A. (2022). Prediction of the Effect of CO_2_ Laser Cutting Conditions on Spruce Wood Cut Characteristics Using an Artificial Neural Network. Appl. Sci..

[3] Kubovský I., Krišťák Ľ., Suja J., Gajtanska M., Igaz R., Ružiak I., Réh R. (2020). Optimization of Parameters for the Cutting of Wood-Based Materials by a CO_2_ Laser. Appl. Sci..

[4] Nagai K., Shimizu K. (2021). Using a High-Power Fibre Laser to Cut Concrete. Appl. Sci..

[5] Seo Y., Lee D., Pyo S. (2020). High-Power Fiber Laser Cutting for 50-mm-Thick Cement-Based Materials. Materials.

[6] Lee D., Seo Y., Pyo S. (2018). Effect of Laser Speed on Cutting Characteristics of Cement-Based Materials. Materials.

[7] Ninikas K., Kechagias J., Salonitis K. (2021). The Impact of Process Parameters on Surface Roughness and Dimensional Accuracy during CO_2_ Laser Cutting of PMMA Thin Sheets. J. Manuf. Mater. Process..

[8] Mushtaq R.T., Wang Y., Rehman M., Khan A.M., Mia M. (2020). State-Of-The-Art and Trends in CO_2_ Laser Cutting of Polymeric Materials—A Review. Materials.

[9] Chen X., Li T., Zhai K., Hu Z., Zhou M. (2017). Using orthogonal experimental method optimizing surface quality of CO_2_ laser cutting process for PMMA microchannels. Int. J. Adv. Manuf. Technol..

[10] Haddadi E., Moradi M., Karimzad Ghavidel A., Karimzad Ghavidel A., Meiabadi S. (2019). Experimental and parametric evaluation of cut quality characteristics in CO_2_ laser cutting of polystyrene. Optik.

[11] Girdu C.C., Gheorghe C. (2022). Simulation of Melting Efficiency in Laser Cutting of Hardox 400 Steel. Materials.

[12] Girdu C.C., Gheorghe C., Radulescu C., Cirtina D. (2021). Influence of Process Parameters on Cutting Width in CO_2_ Laser Processing of Hardox 400 Steel. Appl. Sci..

[13] Mahrle A., Borkmann M., Pfohl P. (2021). Factorial Analysis of Fiber Laser Fusion Cutting of AISI 304 Stainless Steel: Evaluation of Effects on Process Performance, Kerf Geometry and Cut Edge Roughness. Materials.

[14] Son S., Lee D. (2020). The Effect of Laser Parameters on Cutting Metallic Materials. Materials.

[15] Riveiro A., Quintero F., Boutinguiza M., Del Val J., Comesaña R., Lusquiños F., Pou J. (2019). Laser Cutting: A Review on the Influence of Assist Gas. Materials.

[16] Fomin V.M., Golyshev A.A., Orishich A.M., Shulyatev V.B. (2017). Energy balance in high-quality cutting of steel by fiber and CO_2_ lasers. J. Appl. Mech. Tech. Phys..

[17] He Y., Xie H., Ge Y., Lin Y., Yao Z., Wang B., Jin M., Liu J., Chen X., Sun Y. (2022). Laser Cutting Technologies and Corresponding Pollution Control Strategy. Processes.

[18] Buj-Corral I., Costa-Herrero L., Domínguez-Fernández A. (2021). Effect of Process Parameters on the Quality of Laser-Cut Stainless Steel Thin Plates. Metals.

[19] Happonen A., Stepanov A., Piili H., Salminen A. (2015). Innovation Study for Laser Cutting of Complex Geometries with Paper Materials. Phys. Procedia.

[20] Ready J.F. (1997). Industrial Applications of Lasers.

[21] Rämö S. (2004). Effects of Coating on Laser Cuttability of Coated Papers and Boards. Master’s Thesis.

[22] Stepanov A., Saukkonen E., Piili H., Salminen A. (2015). Effect of Moisture Content of Paper Material on Laser Cutting. Phys. Procedia.

[23] Al Drais S., Al Zhraa Ibrahim F., Al Fouderi N., Mamedov A. Design of an automated paper cutting machine. Proceedings of the International Conference on Industrial Engineering and Operations Management.

[24] Yin Z., Xu L. Finite Element Analysis and Optimization Design of Paper Cutter Cutting Blade Based on ANSYS. Proceedings of the International Conference on Robots and Intelligent System (ICRIS 2018).

[25] Piili H. (2015). A Theory of Interaction Mechanism between Laser Beam and Paper Material. Phys. Procedia.

[26] Riley A. (2012). Paper and paperboard packaging. Packaging Technology.

[27] Kirwan M.J. (2005). Paper and Paperboard Packaging Technology.

[28] Malmberg H., Kujanpää V. (2006). Cutting paper—Application report. Ind. Laser Solut. Manuf..

[29] Piili H. (2013). Characterization of Laser Beam and Paper Material Interaction. Ph.D. Thesis.

[30] Hovikorpi J., Laakso P., Malmberg H., Kujanpää V., Miikki N. Laser cutting of paper. Proceedings of the 23rd International Congress on Laser Materials Processing and Laser Microfabrication.

[31] Federle H., Keller S. (1992). Papier schneiden mit Laser. Pap. Kunstst. Verarb..

[32] Malmberg H., Leino K., Kujanpää V. (2006). Laser Cutting of Paper and Board.

[33] Malmberg H., Salminen A., Kujanpää V. Laser cutting of mineral pigment coated papers. Proceedings of the 25th International Congress on Applications of Laser and Electro-Optics, Congress Proceedings.

[34] Stepanov A., Piili H., Salminen A. Color change in laser cutting of paper material. Proceedings of the 29th International Congress on Applications of Lasers and Electro-Optics.

[35] Stepanov A., Piili H., Saukkonen E., Salminen A. Effect of linear cutting energy on coloration of paper in laser cutting of paper material. Proceedings of the 30th International Congress on Applications of Lasers and Electro-Optics.

[36] Spicar-Mihalic P., Toley B., Houghtaling J., Liang T., Yager P., Fu E. (2013). CO_2_ laser cutting and ablative etching for the fabrication of paper-based devices. J. Micromech. Microeng..

[37] Mahmud M.A., Blondeel E.J.M., Kaddoura M., MacDonald B.D. (2016). Creating compact and microscale features in paper-based devices by laser cutting. Analyst.

[38] Mahmud M.A., Blondeel E.J.M., Kaddoura M., MacDonald B.D. (2018). Features in Microfluidic Paper-Based Devices Made by Laser Cutting: How Small Can They Be?. Micromachines.

[39] Nie J., Liang Y., Zhang Y., Le S., Li D., Zhang S. (2013). One-step patterning of hollow microstructures in paper by laser cutting to create microfluidic analytical devices. Analyst.

[40] Pillai S.P., Abhiram B.R., Kumar A.S., Ashish U.S., Harikrishnan G. (2019). Solar powered desktop CNC machine for fabric and paper cutting. IOP Conf. Ser.: Mater. Sci. Eng..

[41] Pagès H., Piombini H., Enguehard F., Acher O. (2005). Demonstration of paper cutting using single emitter laser diode and infrared-absorbing ink. Opt. Express..

[42] Huppert F., Holzl G., Kranz M. (2019). Design Different: Pen and Paper for Laser Cutting. IEEE Pervasive Comput..

[43] Happonen A., Stepanov A., Piili H. (2015). Feasible Application Area Study for Linear Laser Cutting in Paper Making Processes. Phys. Procedia.

[44] Jones B., Nachtsheim C.J. (2013). Definitive screening designs with added two-level categorical factors. J. Qual. Technol..

[45] Jones B., Nachtsheim C.J. (2011). A class of three-level designs for definitive screening in the presence of second-order effects. J. Qual. Technol..

[46] Xiao L., Lin D.K.J., Bai F. (2012). Constructing definitive screening designs using conference matrices. J. Qual. Technol..

[47] Jones B., Nachtsheim C.J. (2017). Effective design-based model selection for definitive screening designs. Technometrics.

